# Mechanical Performance Evaluation of Repair Materials Suitable for Mechanical Pressurizing Equipment for Cross-Sectional Repair of Concrete Box Structures

**DOI:** 10.3390/ma16041472

**Published:** 2023-02-09

**Authors:** Jung-Youl Choi, Sun-Hee Kim, Hyeong Sik Yu, Jee-Seung Chung

**Affiliations:** 1 Department of Construction Engineering, Dongyang University, No. 145 Dongyangdae-ro, Punggi-eup, Yeongju-si 36040, Gyeongsangbuk-do, Republic of Korea; 2 Department of Architectural Engineering, Gachon University, 1342 Seongnamdaero, Sujeong-gu, Seongnam-si 13120, Gyeonggi-do, Republic of Korea; 3 No-Dig Tech., 24, Dunchon-daero 388, Jungwon-gu, Seongnam-si 13403, Gyeonggi-do, Republic of Korea

**Keywords:** mechanical pressurizing equipment, high aluminate cement-mixed mortar, new repair material, pressurization effect, bond performance

## Abstract

This study entailed performance tests to confirm the bond performance of the proposed new repair material and the pressurization effect of the developed mechanical pressurizing equipment. The physical property changes of the new repair material were reviewed by varying the mixing ratio of high aluminate cement (HAC)-mixed mortar. Strength tests were performed according to the mixing ratios of polymer and silica fume to improve the bond performance. To improve water retention, the mixing ratios of the cellulose and nylon fibers were adjusted, and the change in water retention was measured. The proposed repair material mixing ratio yielded the best performance when pressure was applied to the repair surface. Comparing the existing repair materials and the new repair material prepared by adjusting the ratios of HAC-mixed mortar, cellulose fiber, redispersible powder resin, and other factors confirmed that the new repair material has a high bond strength.

## 1. Introduction

In cross-sectional repair methods, polymer-mortar is mainly used for repair and reinforcement. Recently, however, high aluminate cement (HAC), which has excellent rapid hardening and chemical resistance properties, is being increasingly used. A lightweight mortar and thickener are utilized to reduce bond strength degradation due to self-weight in the cross-sectional repair of ceilings and walls.

**Figure 1:**
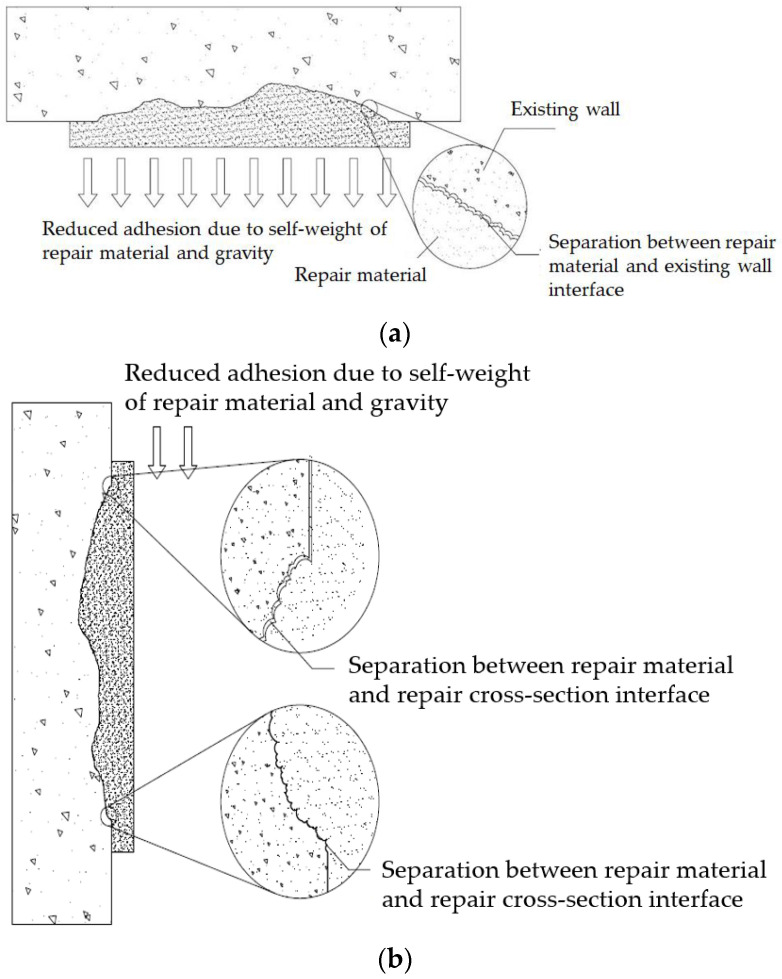
Separation of concrete structure. (**a**) Ceiling separation; (**b**) wall separation.

Cross-sectional repair methods applied to deteriorated concrete cross-sections involve removing the deteriorated concrete and filling the void with repair materials such as epoxy or mortar. However, because of the influence of self-weight and gravity that continuously acts on the repair materials, separation occurs between the new and old materials in the ceiling or wall, as shown in Figure 1 [1]. To reduce the influence of gravity, which is the fundamental cause of reduced bonding strength, studies have been conducted on lightweight materials, additives that increase adhesion to the base, and rapid hardening additives.

Among Korean studies that evaluated the mechanical performance of segment lining in which a waterproofing membrane was deposited through spraying, Kang et al. [2] proposed certain mixing conditions for membrane prototypes. According to the performance test of the segment, the initial crack occurrence was delayed and increased by approximately 34%. Kim et al. [3] evaluated the mechanical performance of concrete after applying a silicate-based surface penetrant. They found that spraying a photocatalyst effectively modifies the concrete surface and allows photocatalytic adsorption. Through a performance evaluation, Lee [4] verified that calcium aluminate cement mortar yielded better mechanical performance than ordinary Portland cement (OPC) mortar. By evaluating the flexural bonding performance of hybrid concrete repair materials, Kim et al. [5] found that increasing the powder resin content increased the bonding strength. Cho et al. [6] conducted performance evaluations to assess the leakage repair materials of two-component adhesive sealing materials; the washout resistance and chemical resistance performance tests demonstrated that the materials’ rate of mass change increased. Won et al. [7] tested the mechanical performance and fire resistance of polymer-modified cementitious composite repair materials to evaluate their applicability in the repair of concrete tunnel structures. They found that the structure was safe when a polymer-modified cementitious composite with a thickness of at least 40 mm was applied to protect it from fires. Lee and Han [8] reduced the cement content in the mortar and applied graphene oxide and porous feldspar. Therefore, it was confirmed that the compressive strength of the mortar was maintained by increasing the bond strength of the hydrate. Through experiments, Lee [9] confirmed the bonding efficiency of the mortar mixed with fly ash. As a result, the strength of the mortar mixed with fly ash differed depending on the age and the effect of a water/binder ratio.

Among non-Korean studies assessing tunnel lining damage, Liu et al. [10] evaluated the structural performance and failure mechanisms through a numerical analysis. They found that the lining could be reinforced through crack grouting and shotcrete support. Zhou [11] proposed a new plate-type anchor structure to reinforce tunnel cracks and experimentally verified the state changes of the plate-short anchor assembly structure in a cracked state. According to their comparison of the bearing capacities of the anchor-reinforced and unreinforced parts of a specimen, reinforcement could improve the bearing by at least 16%. To examine the apparent quality and service performance of self-compacting fair-faced concrete, Xie et al. [12] conducted mechanical tests on specimens with different mixing ratios and proposed appropriate mixing ratios based on the results. Kimura et al. [13] developed a tunnel structure evaluation method and technique to quantitatively evaluate the overall performance of existing railway tunnels. When repairing and reinforcing concrete box structures, the repair material adhesion degrades due to gravity, vibrations, and the surface conditions of the bond surface, making it impossible to ensure repair quality [1,14]. In this study, a repair material was developed that exhibits an improved adhesion performance and chemical resistance compared to existing repair materials. Physical property tests were performed on repair materials suitable for pressurization systems to increase the bond strength by reducing the influence of gravity acting on the target cross-section through pressure. The system uses the physical property, pressure, unlike the existing repair methods that use chemical properties. A physical property test was conducted to ensure the repair material satisfies the principle of improving the repair cross-section and adhesion by continuously maintaining a press-in state. Considering factors such as a delayed hardening reaction due to pressure and the forced moisture discharge expected in the target cross-section due to applied pressure, additives were selected, and their addition ratios were adjusted to calculate the optimal mixing ratio.

## 2. Methodology

### 2.1. Equipment

**Figure 2:**
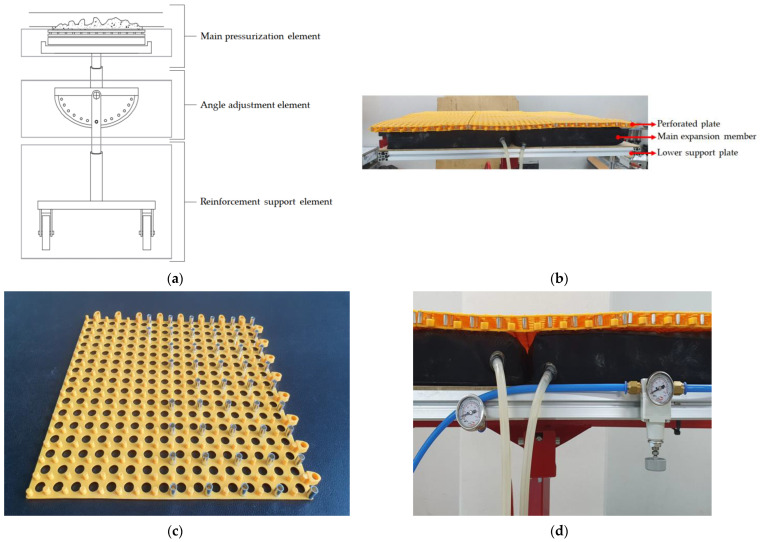
Composition of MPE. (**a**) Detailed diagram of MPE; (**b**) main pressurization part; (**c**) perforated plate of the main pressurization part; (**d**) pressure adjustment part of the main expansion member.

To solve the separation problems represented in Figure 1, this study developed mechanical pressurizing equipment (MPE, Seongnam-si, Republic of Korea), as shown in Figure 2. Figure 2a presents a detailed diagram of the MPE; the reinforcement support consists of easily movable wheels and a plate located below the angle adjustment element and supports and maintains the pressure generated in the main pressurization element. As shown in Figure 2b, the main pressurization element consists of the perforated plate (Figure 2c), main expansion member, and lower support plate. By forming an installation space with a rod that can maintain a gap with the perforated plate, it has sufficient contact with air when pressurizing the repair material. The contact area between the perforated plate and mortar was made of a flexible material, ensuring a uniform pressure was applied to the irregular repair surface. A pressure gauge and pressure controller were attached to the main expansion member, ensuring a constant pressure was applied, as shown in Figure 2d. 

### 2.2. Materials

The main component of HAC is calcium aluminate. Thus, unlike OPC, whose main component is calcium silicate, in addition to being used as a hydraulic material, HAC develops early strength when used with OPC by promoting hydration. HAC can also produce an expansion effect when combined with CaSO_4_, thus making it effective for controlling shrinkage cracks mainly caused by drying and shrinkage. Furthermore, unlike OPC, HAC does not generate portlandite (Ca(OH)_2_) during the hydration reaction; therefore, CaSO_4_·2H_2_O is not generated in the deterioration mechanism through sulfates or biochemical corrosion, and expansion and erosion do not occur. Therefore, HAC is extensively used in cross-sectional repair materials to compensate for shrinkage, promote solidification, and develop early strength. Particularly, HAC is known to have excellent chemical resistance in biochemically corrosive environments, depending on the amount of HAC used. Accordingly, to obtain excellent corrosion resistance and increase early stiffness and crack control, this study conducted mechanical performance tests on mortars containing HAC in accordance with KS F 4042 [15].

### 2.3. Mortar Mixtures

#### 2.3.1. Mortar Mixtures according to HAC Content

Table 1 lists the mortar mixtures used to identify the mechanical properties and chemical resistance of the mortar according to the amount of HAC used. Owing to the strength of the mortar varying according to the amount of HAC used, the mechanical properties of the mortar were confirmed. The water–binder ratio of the mortar was 0.4, and the binder:fine aggregate ratio was 1:2.7. The following mixing order was applied to prepare the mortar: The OPC and/or HAC and fine aggregate were placed in a 10 L mixer and dry mixed at 30 to 40 rpm for 2 min, after which the mixing water was added and mixed at 70 to 80 rpm for 3 min.

#### 2.3.2. Mortar Mixtures according to Fiber Content

Cellulose fibers were added at 1% and 2%. For comparison, nylon fiber, an artificial fiber, was also added at 1% and 2%. The physical properties of the mixtures were examined. Table 2 lists the mixtures of these mortars.

### 2.4. Flow Test

A flow test was performed in accordance with KS L 5105 [16] to confirm the flow characteristics of the mortar according to the HAC mixing ratio.

### 2.5. Compressive Strength

Compressive strength tests were conducted on the mortar according to the HAC mixing ratios in accordance with KS L 5105 [16]. For each mixing ratio listed in Table 1, three 50 mm cube mortar specimens were prepared and then cured in water. Their compressive strengths were then measured at the ages of 3, 7, and 28 days.

### 2.6. Acid Resistance

(1) Investigation of mortar appearance

**Figure 3:**
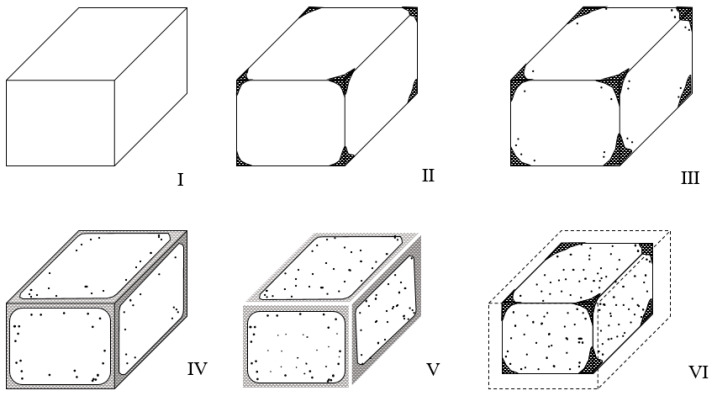
Appearance grades of mortar eroded by acid. (**I**) Negligible; (**II**) very small deterioration; (**III**) detectable deterioration; (**IV**) delamination and small mass loss; (**V**) considerable mass loss; (**VI**) almost failure.

To evaluate the acid resistance of the mortar specimens, ninety-six 50 mm cube specimens were immersed in fresh water, a 5% sulfuric acid solution, and a 10% sulfuric acid solution for 14 and 28 days, after which the appearance of the specimens was investigated. As shown in Figure 3, the appearance standard and damage conditions through chemical erosion were classified into six grades, according to a previous study [17].

(2) Compressive strength according to acid or water immersion

To evaluate the acid erosion resistance of the mortar specimens, the compressive strength of the mortar specimens immersed in the acid solutions and freshwater was measured to calculate the compressive strength loss (*CSL*), as shown in Equation (1) [14].

(1) $$CSL\;(\%)=\frac{C_{W}-C_{S}}{C_{W}}\times 100$$

where $C_{W}$  is the compressive strength of the mortar specimen cured in freshwater (MPa), and $C_{S}$  is the compressive strength of the mortar specimen immersed in the test solution (MPa).

(3) Mass loss of mortar

The age-specific masses of the mortars immersed in the two acid solutions were measured, and their losses compared to the initial masses were measured using Equation (2) [12].

(2) $$weight\;loss\;(\%)\;=\frac{W_{i}-W_{t}}{W_{i}}\times \;100\;$$

where $W_{i}$  is the initial mass (g) of the mortar specimen before immersion in the acid solution, and $W_{t}$  is the mass (g) of the mortar specimen after an elapse of time *t* after immersion in the acid solution.

### 2.7. Flexural Strength

The flexural strength measurements were determined based on the quality evaluation criteria presented in KS F 4042 [15] for the mortar according to the additive mixing ratio.

### 2.8. Bond Strength

The mortar bond strength measurements of twenty-seven Ø40 × 30 mm specimens attached to a base plate were obtained according to the additive mixing ratio.

### 2.9. Water Retention Test

**Figure 4:**
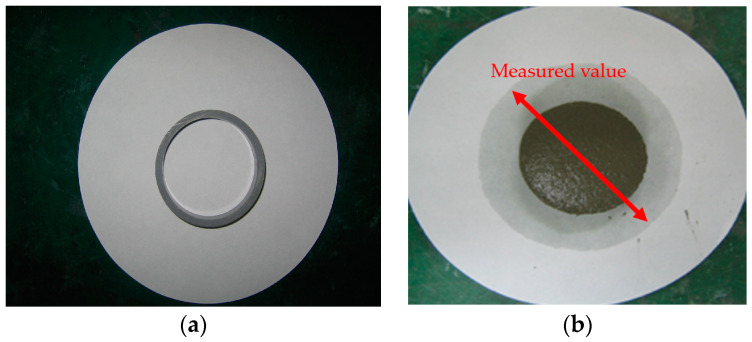
Water retention test. (**a**) Before the test; (**b**) after the test.

To measure the water retention of the thickener, the mortars prepared according to the mixtures listed in Table 2 were placed in a ring with a diameter of 50 mm on absorbent paper and left for 20 min, after which the diameter of the water absorbed by the absorbent paper was measured. Equation (3) was used to calculate the water retention, and Figure 4 shows the water retention test [16].

(3) $$Water\;retention\;(\%)=\frac{50}{measured\;value\;(mm)}\times 100$$

### 2.10. Specific Gravity and Air Content Tests

To perform the specific gravity test, the slurry mortar was compacted 25 times in two layers in a specific gravity cup, after which the weight was measured, and the specific gravity was calculated. The air content was measured using a general pressure method in accordance with KS F 2421 [18].

### 2.11. Length Change Ratio

The change in length was measured using a length change tester and then converted to a change ratio.

## 3. Mechanical Performance of Repair Materials according to the Additive Mixing Ratio

### 3.1. Flow Test

**Figure 5:**
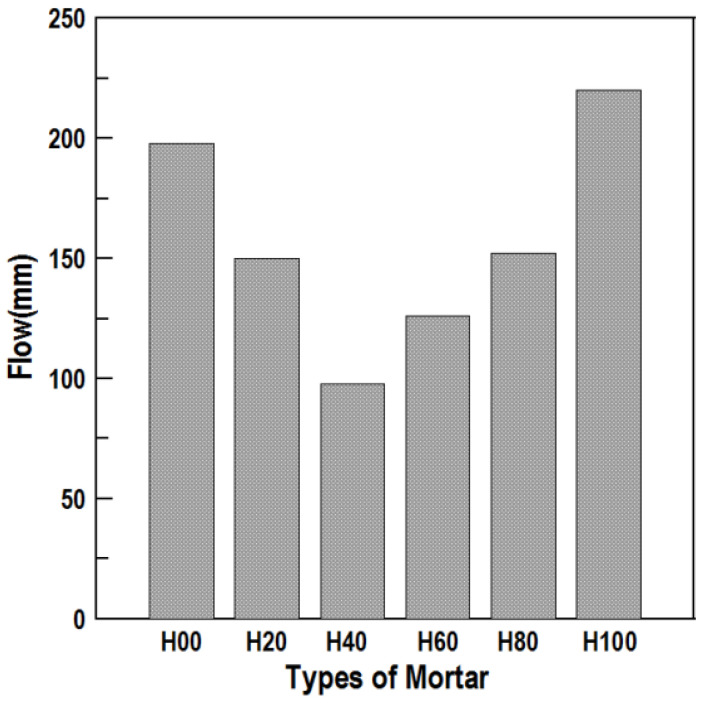
Flow according to HAC mixing ratios.

According to the flow test results, as shown in Figure 5, the flow values of H20, H40, H60, and H80 were smaller than that of H00, and the flow value of H100 was larger than that of H00. Though it seems necessary to consider the amount of HAC-mixed mortar used in terms of fluidity based on the test results, a superplasticizer was applied in the actual repair material mixture, and the W/C was adjusted according to workability and strength standards. Therefore, the effect of the fluidity according to the HAC mixing ratio on the new repair material is judged to be very limited.

### 3.2. Compressive Strength according to the HAC Mixing Ratio

**Figure 6:**
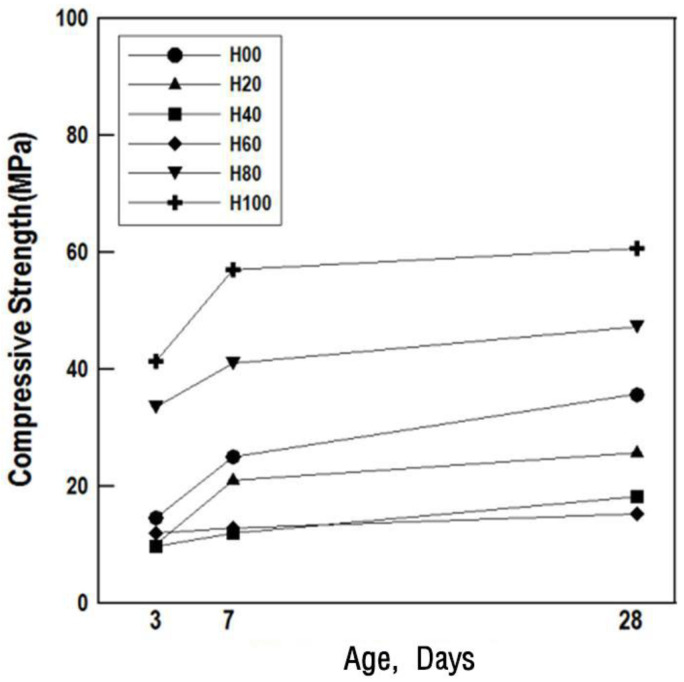
Compressive strength of the alumina cement-mixed mortars.

**Figure 7:**
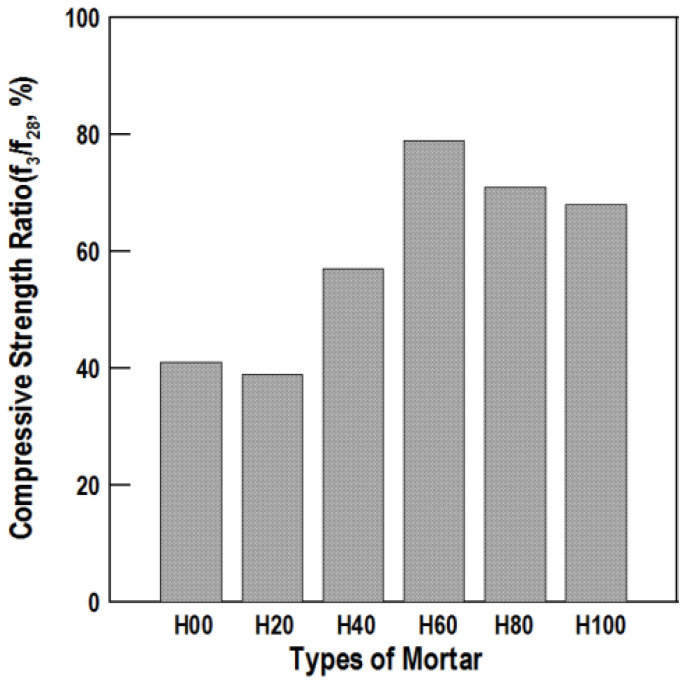
Compressive strength ratio of the mortars.

Figure 6 and Figure 7 show the compressive strength values and ratios, respectively, by mortar specimen age according to the HAC mixing ratio.

The 3-day compressive strength of H00 was similar to that of H20, H40, and H60, and the high strength development in H80 and H100. At 28 days, the compressive strength of H00, H20, H40, H60, H80, and H100 was 35.4, 25.1, 18.6, 14.9, 47.7, and 60.3 MPa, respectively. According to the compressive strength tests, the compressive strength ratio was large when the mixing ratio of HAC was at least 60%. Therefore, a mixing ratio of at least 80% is judged to be appropriate to obtain a high compressive strength in HAC-mixed mortar.

### 3.3. Acid Resistance

(1) Investigation of mortar appearance

**Figure 8:**
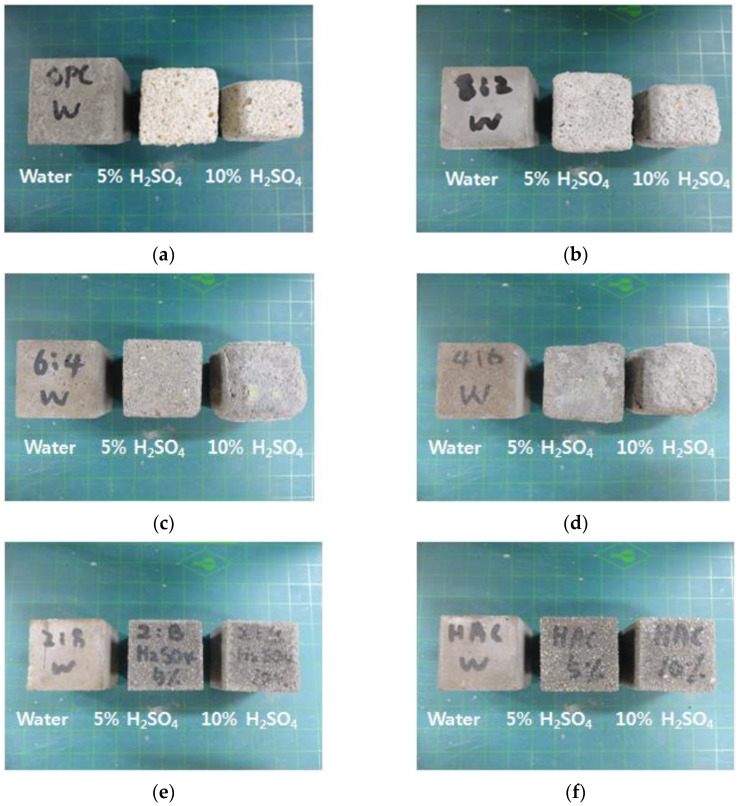
Appearance of the mortars (28 days). (**a**) H00; (**b**) H20; (**c**) H40; (**d**) H60; (**e**) H80; (**f**) H100.

**Figure 9:**
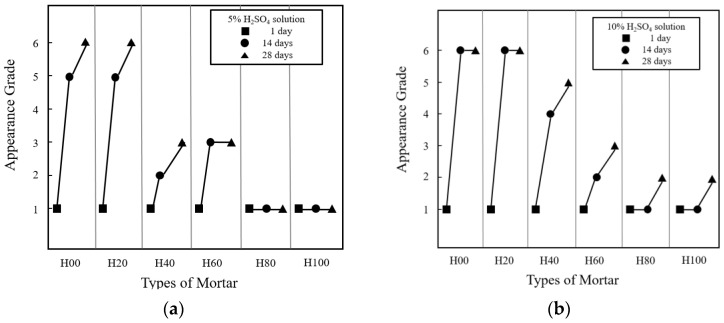
Appearance grades of the mortars. (**a**) 5% H_2_SO_4_; (**b**) 10% H_2_SO_4_.

Figure 8 shows the appearances of the eroded HAC-mixed mortars. The appearance grades of the six types of mortar are categorized by acid solution concentration and presented in Table 3 and Figure 9. Figure 8 shows that, compared to the H00 mortar cured in water, the H00 mortar immersed in the acid solution was significantly eroded, resulting in prominent section loss and generation of reaction products. Moreover, according to a comparison with H00 mortar immersed in 5% H_2_SO_4_ solution, as presented in Table 3 and Figure 3, the mortar immersed in the 10% HSO solution had already reached appearance grade VI at 14 days and erosion to the point of failure occurrence. The H00 mortar immersed in 10% H_2_SO_4_ solution exhibited more than 40% section loss, demonstrating that the degree of erosion varied with the acid solution concentration.

The appearances of the acid-eroded H80 and H100 mortars showed excellent acid erosion resistance compared to the mortars mixed with a small amount of HAC. Particularly, even when immersed in 10% H_2_SO_4_, there was barely any performance degradation due to acid erosion. The HAC-mixed mortars have excellent acid resistance because alumina gel, whose main component is Al_2_(OH)_3_, is formed, creating a protective layer against the acid solution. In contrast, calcium hydroxide (Ca(OH)_2_) generates when general OPC is hydrated and gypsum (CaSO_4_·2H_2_O) is dehydrated, and has a weak structure due to its porosity.

(2) Compressive strength according to acid or water immersion

**Figure 10:**
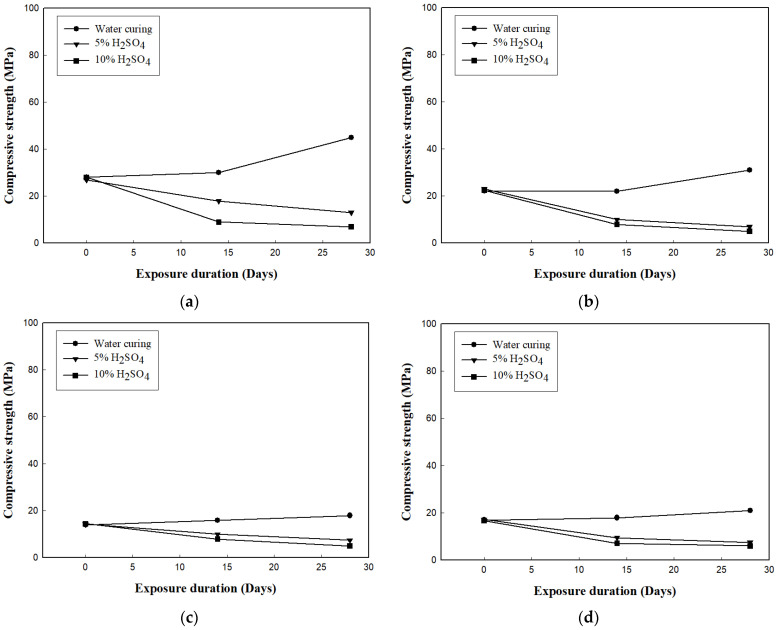

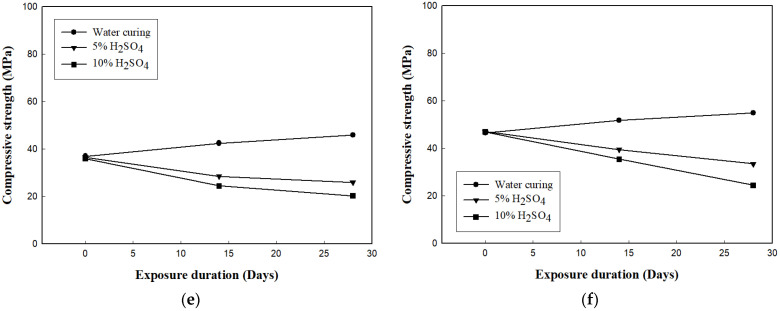
Change in the compressive strength of the mortars after acid or water immersion. (**a**) H00; (**b**) H20; (**c**) H40; (**d**) H60; (**e**) H80; (**f**) H100.

Figure 10 shows the change in compressive strength of the cement mortars.

Figure 10a shows the change in the compressive strength of H00. For the mortar cured in water, the compressive strength increased as the exposure period increased. However, the compressive strength of the mortar immersed in the acid solution decreased as the exposure period increased, and the mortar immersed in 10% H_2_SO_4_ solution exhibited a greater decrease in compressive strength than the mortar immersed in 5% H_2_SO_4_ solution. At 28 days, the H00 mortar cured in water showed a compressive strength of approximately 46.0 MPa, whereas the compressive strength of the mortars immersed in 5% and 10% H_2_SO_4_ solutions was 12.9 and 6.2 MPa, respectively.

Figure 10b shows the change in compressive strength of H20, which had a similar trend to that of H00. Figure 10c,d shows the change in compressive strength due to the acid erosion of the H40 and H60 mortars, respectively. The compressive strengths of the mortars exposed to 5% and 10% H_2_SO_4_ cured in water were similar. However, the change in compressive strength of H80 (Figure 10e) showed a relatively small decrease in strength due to acid erosion compared to the mortars mixed with a small amount of HAC. Figure 10f shows the change in compressive strength of H100, which exhibited the best acid erosion resistance among the six mortar types.

(3) Mass loss of mortar

**Figure 11:**
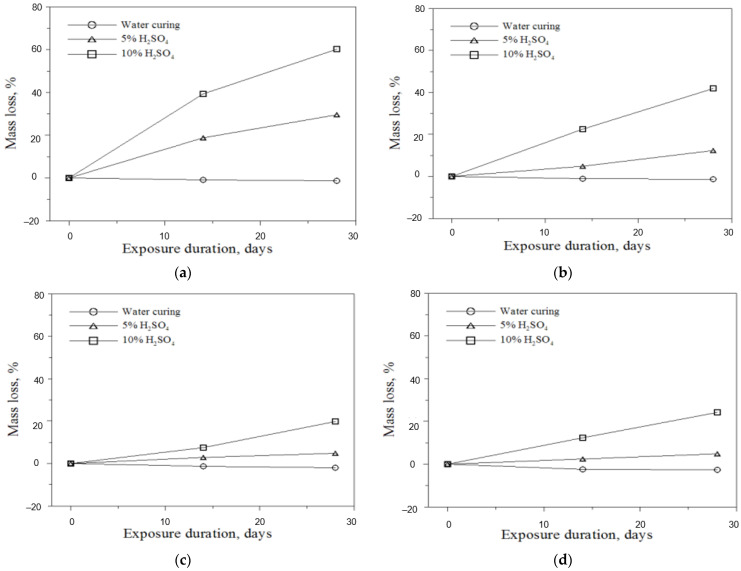

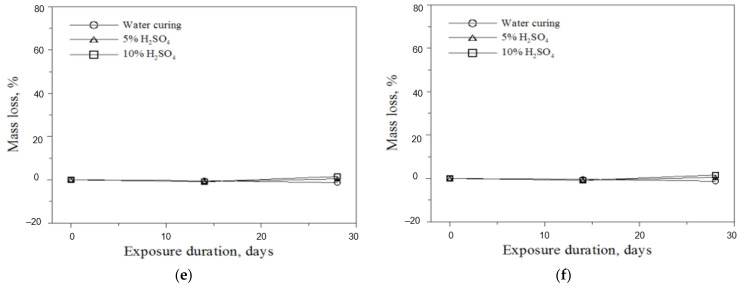
Mass loss of mortar. (**a**) H00; (**b**) H20; (**c**) H40; (**d**) H60; (**e**) H80; (**f**) H100.

Figure 11 shows the change in masses by the age of the acid-eroded mortars. The mass loss at 14 and 28 days after immersing H00 in 5% and 10% H_2_SO_4_ solutions was compared with the mass change of the mortar cured in water; the results are shown in Figure 11a. According to the results, the mass loss increased as the concentration of the immersion solution increased. At 28 days, the mass loss of the mortar immersed in the 5% H_2_SO_4_ solution was 29.5% and that of the mortar immersed in the 10% H_2_SO_4_ solution was 60.2%. Figure 11b shows the mass loss due to the acid erosion of H20. The mass loss was smaller than that of the H00 mortar. Figure 11c,d show the mass loss of H40 and H60, respectively; as indicated, the mass loss due to acid erosion tended to decrease as the replacement rate increased. The mass loss of H80, as shown in Figure 11e, was similar to that of the mortar cured in water regardless of the H_2_SO_4_ solution concentration. The H100 mortar in Figure 11f shows a similar trend. These results confirmed that the performance of the HAC-mixed mortar exposed to an acid erosion environment was excellent when the replacement rate of the HAC-mixed mortar was at least 80%. In this test, it was confirmed that the chemical resistance increased in proportion to the amount of HAC used. For the repair material developed in this study, securing resistance to biochemical corrosion is an important factor in the case of sewage boxes, which are mainly applied. The reason why 100% HAC binder cannot be used is that it should be mixed with OPC for controlling the setting time of the repair material.

## 4. Mechanical Performance of Repair Materials according to the Additive Mixing Ratio

### 4.1. Performance Evaluation of Mortar

Based on the test results according to the HAC content, the ratio of OPC:HAC was set to 20:80 for the binder. The polymer and silica fume were increased in 2% increments from 2% to 6% while examining the physical properties. Table 4 lists the mixing ratios of the polymer and silica fume.

### 4.2. Compressive Strength

**Figure 12:**
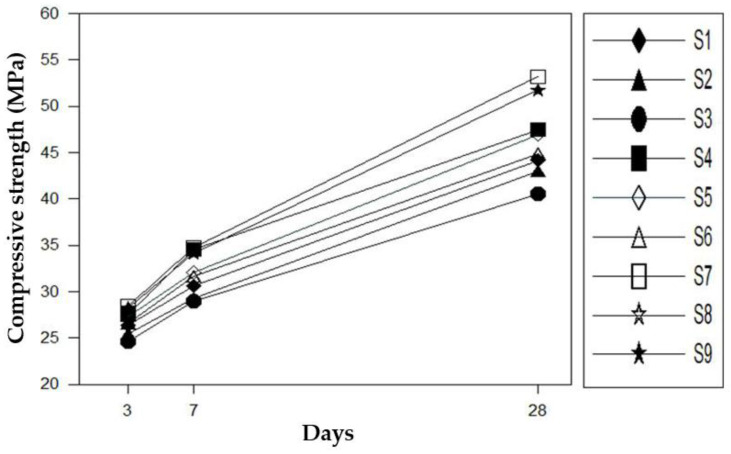
Compressive strength test results.

The compressive strength of the mortar according to the additive mixing ratio was measured according to the KS L 5105 test method [16]; the results are shown in Figure 12. As shown in Figure 12, the compressive strength increased as the silica fume content increased. In the case of mortars of S7–S9 mixed with 6% silica fume, the compressive strength at 28 days showed an enhanced effect of 45 MPa or more. As the amount of binder and the mixing ratio of silica fume increased, the strength became higher.

This is because silica fume is an ultra-fine particle; therefore, it evenly penetrates the pores of the repair material [19]. Conversely, the compressive strength decreased as the polymer content increased. Although the polymer improved the physical performances, such as adhesion and flexural strength, by forming a film in the cement matrix, this film inhibited the hydration reaction, thus reducing the compressive strength.

### 4.3. Flexural Strength

**Figure 13:**
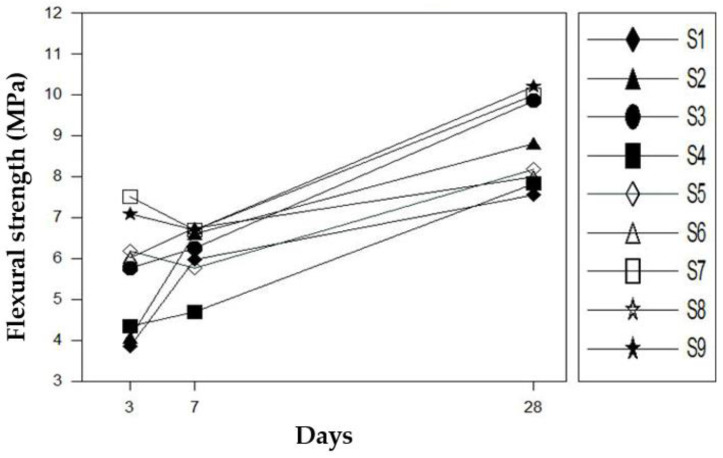
Flexural strength test results.

Figure 13 shows the flexural strength measurements of the mortar according to the additive mixing ratio based on the quality evaluation criteria presented in KS F 4042 [15].

The flexural strength increased as the polymer and silica fume contents increased. This is because the polymer increases the ductility of the mortar by enhancing the bond force between hydrates, while the silica fume raises the mortar’s density and thus increases its physical strength, thereby increasing the flexural strength according to the compressive strength. Therefore, in the case of flexural strength, it was confirmed that it increased as the content of polymer and silica fume increased.

### 4.4. Bond Strength

**Figure 14:**
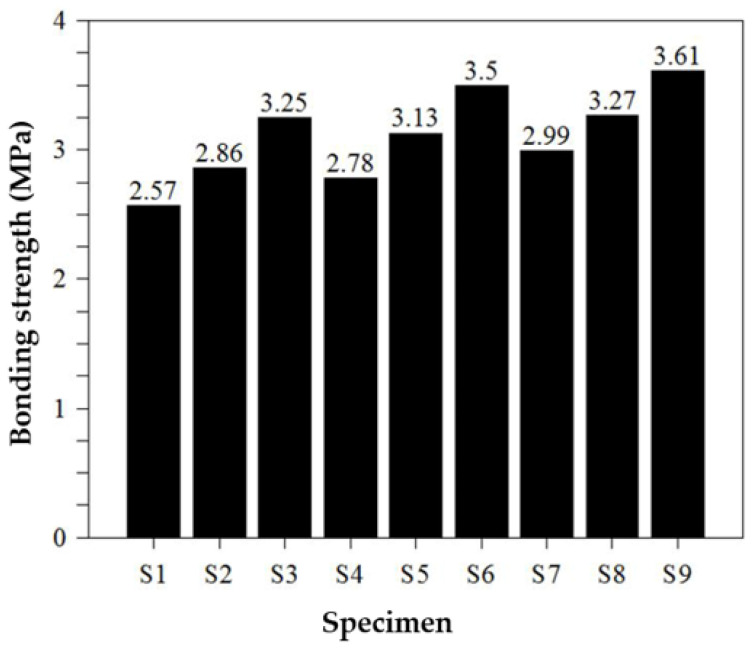
Bonding strength test results.

Figure 14 shows the mortar bond strength measurements of twenty-seven Ø40 × 30 mm specimens attached to a base plate according to the additive mixing ratio. Since the polymer, which contributes the most to the bond strength, has the characteristics of an organic adhesive, it can have a different adhesion mechanism from inorganic binders during dry hardening. Thus, the bond performance to the adherend is attained by organic and inorganic mechanisms, and for silica fume, this effect can be obtained by increasing the overall physical strength. When silica fume was applied in the bond strength test, all strength factors increased proportionally to the quantity added, regardless of the strength measured. However, since the water absorption content increased due to the characteristics of the ultrafine particles, a high-performance superplasticizer must be added. Therefore, the silica fume content was set at 4% in this study, in which case approximately 0% to 1% of superplasticizer should be added and mixed. In the case of organic polymers, there is a limit to their use in designing materials or economical repair materials that contribute decisively to not only flexible strength but also bonding strength. In addition, as the amount of polymer used increases, the compressive strength decreases; thus, it is considered that 2% of the polymer is appropriate.

## 5. Water Retention of Repair Materials according to the Fiber Mixing Ratio

### 5.1. Cellulose Fibers

To develop suitable repair materials for pressurization systems, the interactions between the materials and construction methods and the effect of high-efficiency cellulose fibers were confirmed through tests. Cellulose, a natural fiber, was used for the fiber component in the repair material. Table 5 lists the physical properties of the cellulose.

### 5.2. Investigation according to the Fiber Type and Content Added

Owing to the design of a repair material, the use of cellulose fibers as a thickening stabilizer other than a thickening agent was reviewed. Additives such as thickeners impart viscosity to the mortar and prevent the free movement of moisture inside the mortar. Excessive use of a thickener causes excessive adhesion; thus, an appropriate amount should be used. Unlike this, the cellulose fiber releases the moisture trapped inside the capillary by the external force of the pressurization system.

The repair material to be applied to the pressurized system should maintain proper viscosity and adhesiveness in the form of the repair surface at the beginning of construction and not flow down.

Table 6 shows the images of the mortar shapes and lists the water retention measurements according to the fiber type and content added. The water retention of the mortar with 1% cellulose was measured at 66.7%, and that of the mortar with 2% cellulose was measured at 75.8%, indicating high water retention performance.

Cellulose is a natural fiber extracted from pulp, which contains mixed water through capillaries in the fiber to prevent evaporation of moisture and dryness of the surface of the mortar, thereby securing adherence and shape retention performance when placing repair materials.

### 5.3. Water Discharge by the Pressurization System

**Figure 15:**
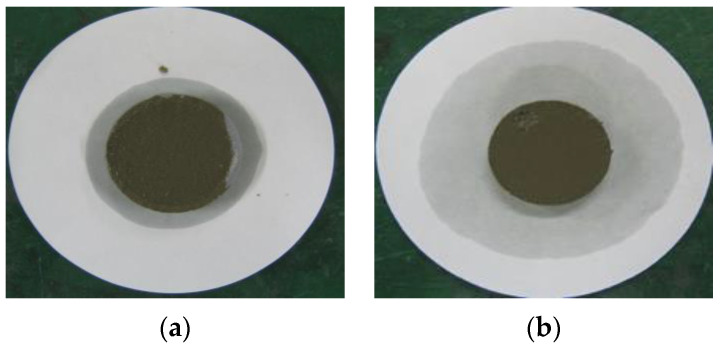
Change in water retention of a developed repair material before and after pressurization. (**a**) Before pressurization; (**b**) after pressurization.

Although cellulose fibers have water retention properties, when the repair material is deposited and applied with pressure through the pressurization system, the moisture in the capillaries is instantaneously discharged to the outside, as shown in Figure 15. When pressurized by the pressurization system, surplus mixed water inside the repair material is rapidly discharged to the outside through the pressurization perforated plate; hence, as the mortar is compressed, the bond performance and density are expected to increase through pressurization.

**Figure 16:**
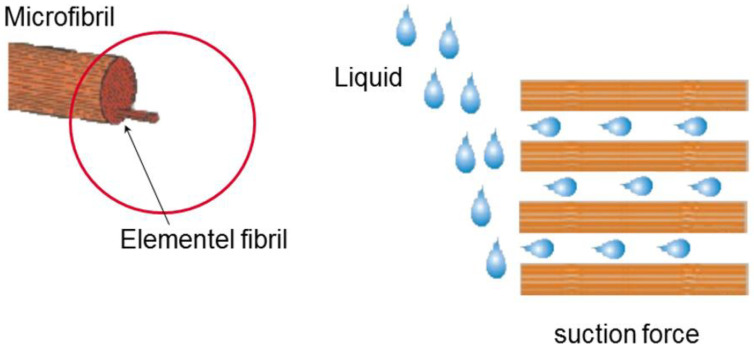
Capillary action of cellulose fibers.

For the material mixed with 1% cellulose, before pressurization, water retention was excellent to the extent that moisture inside the repair material could not permeate into the absorbent paper. After pressurization, however, the capillary action of the cellulose fibers occurred through external pressure, as shown in Figure 16, and the moisture in the capillaries was rapidly discharged to the outside and permeated into the absorbent paper.

## 6. Performance Evaluation of the New Repair Material

### 6.1. New Repair Material

Table 7 lists the optimal mixing ratios of the repair materials prepared by mixing HAC, polymer, silica fume, and cellulose fibers based on the test results in Section 3, Section 4 and Section 5.

For the performance evaluation of repair materials, three variables were set: A cementitious repair material containing polymers mainly used for cross-sectional repair (Type A); a repair material containing fiber reinforcement (Type B); and the repair material proposed in this study (Type C).

### 6.2. Flow Test

**Figure 17:**
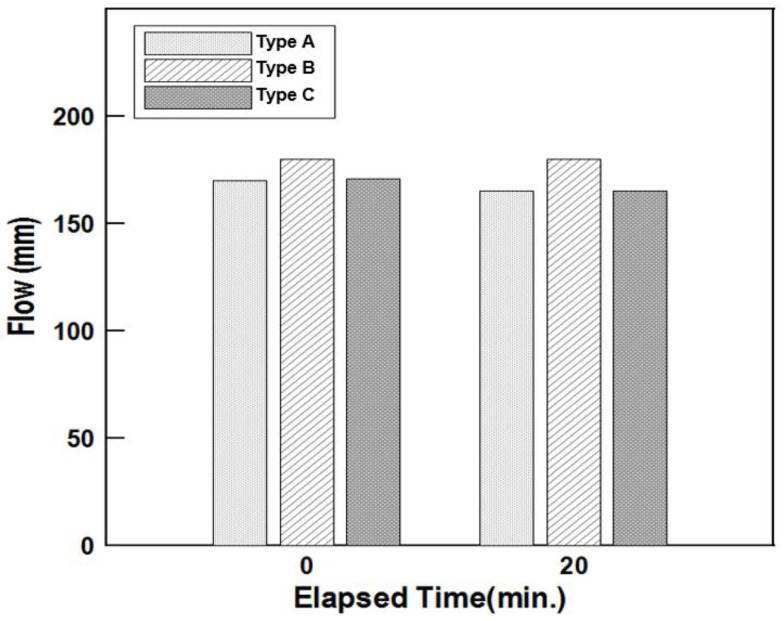
Flow according to repair material type.

The flow value of the cement mortars was measured in accordance with KS L 5105 [16]. Each repair material was measured three times, and the average was converted to a numerical value, the results of which are shown in Table 8 and Figure 17.

The flow values at 0 and 20 min were measured and compared to evaluate the workability of the mortar, as shown in Figure 17. Although the initial flow values did not differ significantly between the repair materials, there were differences in the flow values measured at 20 min. The flow ratio, which can serve as a measure of the duration that the workability is maintained, is obtained by dividing the 20 min flow by the 0 min flow. The flow ratios of the Type A and Type C repair materials were nearly identical at 97%, while the Type B repair material showed the highest workability maintenance performance at 100%. Notably, the difference was not significant.

### 6.3. Specific Gravity and Air Content Tests

**Figure 18:**
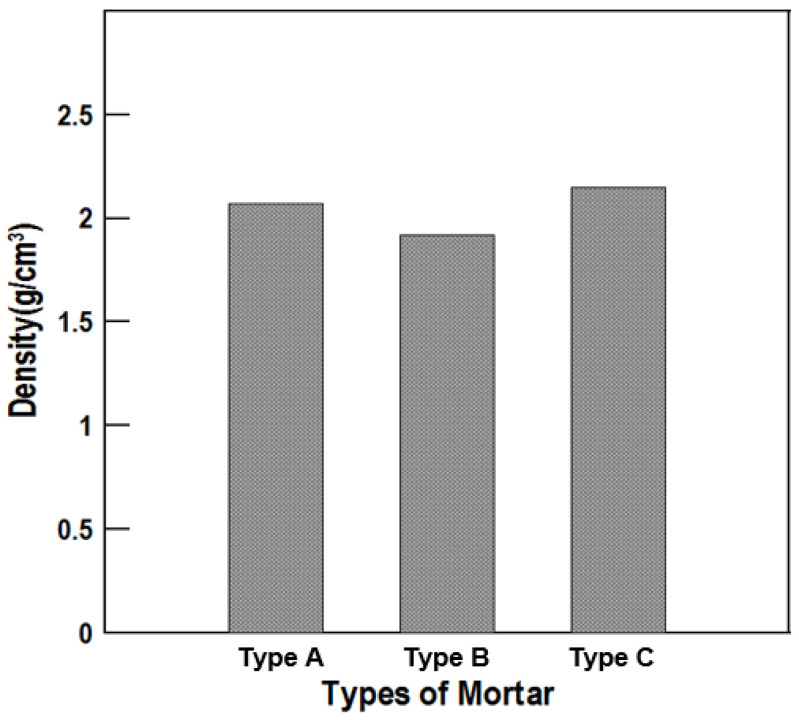
Specific gravity of the repair materials by type.

**Figure 19:**
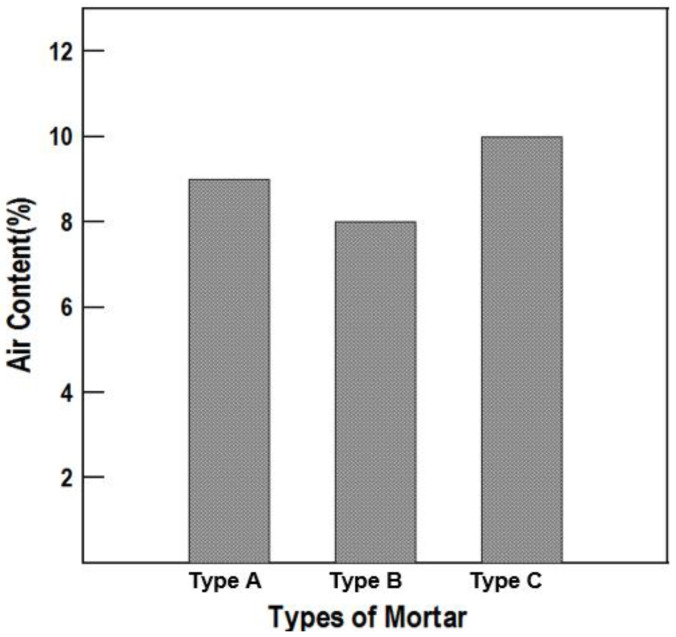
Air content of the repair materials by type.

The results are shown in Table 9 and Figure 18 and Figure 19. In Figure 19, which presents the air content measurements, when an appropriate air content was contained in the mortar, the workability generally tended to improve due to the ball-bearing effect between the pores. The durability was improved by the air bubbles contained in the material, and the air content of each repair material was between 8% and 10%, showing no significant difference. According to the specific gravity test, Type C showed the highest specific gravity.

### 6.4. Length Change Ratio

**Figure 20:**
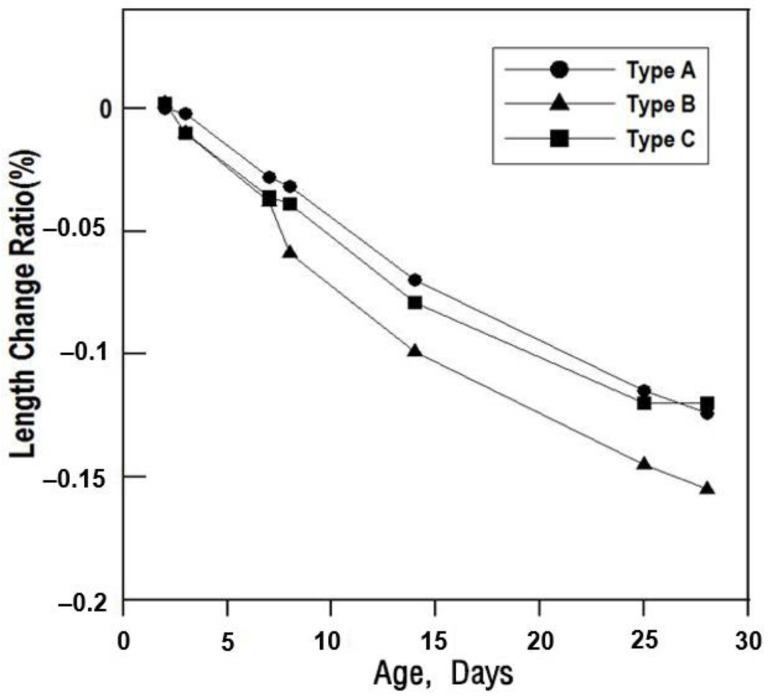
Length change of the specimens by repair material type.

Figure 20 shows the length change results that can confirm the change ratio of shrinkage and expansion of cross-sectional repair materials. All the repair materials exhibited a tendency to shrink regardless of type.

This is a general property of inorganic materials; if the shrinkage rate is constant or does not change significantly, no major problems will occur. If it shrinks significantly during hardening, then irregularities, cracks, lifts, etc. tend to appear; this leads to extremely poor constructability of the top coating and leakage, potentially causing defects in the top coating due to cracks.

According to the test results, the Type B repair material rapidly shrunk after seven days. Although the shrinkage rate was relatively large in the Type A and Type C repair materials, it proceeded at a constant rate, and thus, the length change ratio of the new repair material was judged to be constant.

## 7. Conclusions

When repairing and reinforcing concrete structures, the adhesion of the repair material degrades due to gravity, vibrations, and the surface conditions of the bonding surface. This makes it impossible to secure the necessary repair quality. To solve this problem, this study developed MPE that improves the bonding strength by pressurizing the repair cross-section of the concrete box structure. An HAC-mixed mortar was selected as a material that can ensure acid resistance. The following performance tests results were used to develop a repair material suitable for the developed MPE:

- The flow, compressive strength, and acid resistance tests used to determine the mixing ratio of HAC indicated that the chemical resistance increased proportionally to the amount of HAC used.
- Corrosion resistance must be ensured for the new repair material. Thus, an acid immersion test was conducted which indicated that a high HAC content of approximately 80% of the total binder was suitable.
- The strength tests of the repair materials according to the additive mixing ratios indicated that the performance improved as the content of polymer and silica fume increased.
- The water retention tests of the repair materials according to the fiber mixing ratio indicated that high water retention was ensured when cellulose was added. The water retention of the repair material through the MPE was excellent when 1% cellulose was added.

Based on these materials test results, and considering the properties of general cross-sectional repair materials and applicability of pressurization environments, the optimal mixture of the repair material for the developed MPE was determined to be 6% OPC, 24% HAC, 55% sand, 2% polymer, 4% silica fume, 1% cellulose fiber, and 8% other.

Basic tests were conducted on two existing repair materials and the new repair material applied to the MPE. Compared to the existing repair materials, the new repair material showed a similar flow ratio and air content, high specific gravity, and low length change ratio, indicating excellent resistance to cracking and lifting.

To apply the repair material proposed in this study to the cross-section of a concrete box structures in practice, future studies will analyze the properties of the MPE and conduct bond strength tests for each pressurization condition.

## Figures and Tables

**Table 1:** Mortar mixtures.

Mixtures	W (g)	OPC (g)	HAC (g)	Sand (g)
H00	200	500	-	1350
H20		400	100	
H40		300	200	
H60		200	300	
H80		100	400	
H100		-	500	

**Table 2:** Mixtures of cellulose-added mortars.

Specimen	Mixtures	Binder (H80)	Sand	Polymer (P)	Silica Fume (HD)	Fiber
A	P2%+ HD4%	30	55	2	4	No fiber added
B						1% cellulose
C						2% cellulose
D						1% nylon fiber
E						2% nylon fiber

**Table 3:** Appearance grades of the mortars eroded by acid.

Mortars	5% H_2_SO_4_		10% H_2_SO_4_	
	14 Days	28 Days	14 Days	28 Days
H00	V	Ⅵ	Ⅵ	Ⅵ
H20	V	Ⅵ	Ⅵ	Ⅵ
H40	Ⅱ	Ⅲ	Ⅳ	V
H60	Ⅲ	Ⅲ	II	Ⅲ
H80	I	I	I	Ⅱ
H100	I	I	I	Ⅱ

Ⅰ: Negligible (section loss owing to erosion: 0–3%). II: Very small deterioration (section loss owing to erosion: 3–5%). III: Detectable deterioration (section loss owing to erosion: 5–10%). IV: Delamination and small mass loss (section loss owing to erosion: 10–20%). V: Considerable mass loss (section loss owing to erosion: 20–40%). VI: Almost failure (section loss owing to erosion: More 40%).

**Table 4:** Mixing ratios of polymer and silica fume.

Specimen	Mixtures	Binder (H80)	Sand	Filler	Polymer (P)	Silica Fume (HD)
P	P2%	30	55	13	2	0
S1	P2% + HD2%			11	2	2
S2	P4% + HD2%			9	4	
S3	P6% + HD2%			7	6	
S4	P2% + HD4%			9	2	4
S5	P4% + HD4%			7	4	
S6	P6% + HD4%			5	6	
S7	P2% + HD6%			7	2	6
S8	P4% + HD6%			5	4	
S9	P6% + HD6%			3	6	

**Table 5:** Physical properties of cellulose fiber.

Cellulose Content (%)	Ave Fiber Length (μm)	Ave Fiber Thickness (μm)	Bulk Density (g/L)
90	500	35	70~100

**Table 6:** Water retention results according to added fiber content.

		A	B	C	D	E
Change in water retention		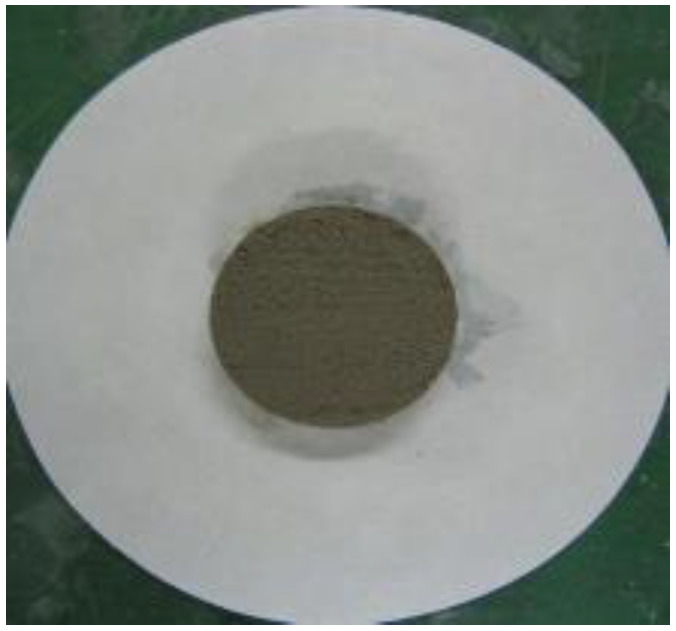	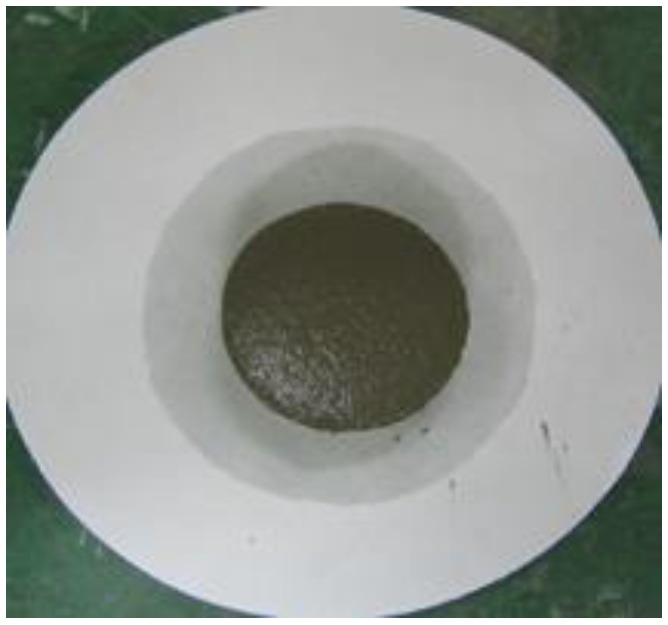	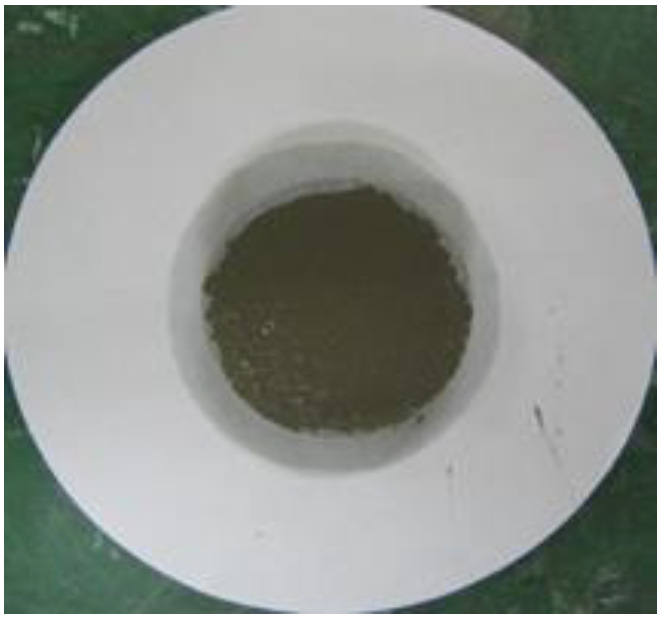	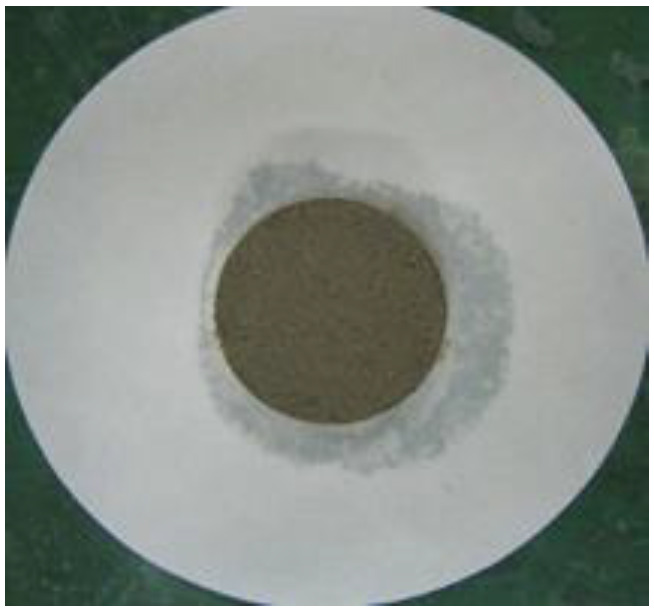	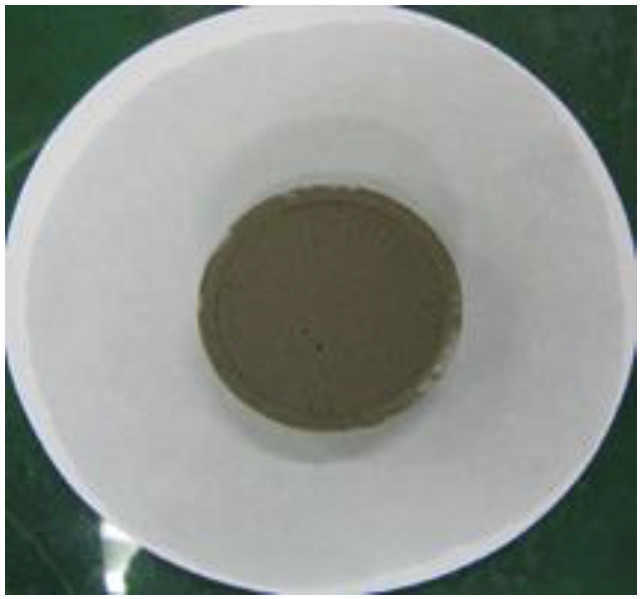
Water retention	mm	NA	75	66	NA	141
	%	0	66.7	75.8	0	35.5

**Table 7:** Materials and mixing ratios used for the new repair materials.

Category	OPC	HAC	Sand	Polymer	Silica Fume	Cellulose Fiber	etc.
Composition ratio (%)	6	24	55	2	4	1	8

**Table 8:** Flow test results.

		Type A	Type B	Type C
Flow (mm)	0 min	170	180	171
	20 min	165	180	165
Flow ratio (%)		97	100	97

**Table 9:** Specific gravity and air content test results.

	Type A	Type B	Type C
Specific gravity	2.07	1.92	2.15
Air content (%)	9	8	10

## Data Availability

http://dongyang.dcollection.net/public_resource/pdf/200000344867_20230209160529.pdf, accessed on 1 February 2023.
